# Tunable Fe_3_O_4_ Nanorods for Enhanced Magnetic Hyperthermia Performance

**DOI:** 10.1038/s41598-020-65095-w

**Published:** 2020-05-20

**Authors:** Yongxiu Yang, Mengwei Huang, Jinmei Qian, Daqiang Gao, Xiaolei Liang

**Affiliations:** 1 Department of obstetrics and gynecology, The First Hospital of Lanzhou University, Key Laboratory for Gynecologic Oncology, Gansu Province, China; 2 Key Laboratory for Magnetism and Magnetic Materials of MOE, Key Laboratory of Special Function Materials and Structure Design of MOE, Lanzhou University, Lanzhou, 730000 P. R. China

**Keywords:** Biophysics, Cancer

## Abstract

Magnetic hyperthermia is one of the most promising techniques for treating gynecological cancer, where magnetite (Fe_3_O_4_) is the most common nanomaterial used as a magnetic hyperthermia agent. Here, we demonstrate that optimal Fe_3_O_4_ nanorods (NRs) can act as a magnetic hyperthermia agent with higher specific absorption rate (SAR), which is mostly attributed to their enhanced surface anisotropy. As a result, Fe_3_O_4_ NRs could effectively hinder the growth of gynecological cancer cells in nude mice models, again demonstrating its good magnetic heating properties. These results provide a powerful basis for the development of an ideal magnetic hyperthermia agent with enhanced SAR, thereby effectively treating gynecological cancer in clinical practice.

## Introduction

Currently, gynecological cancers are seriously affecting women’s health and safety due to environmental pollution and poor eating habits^1–5^. Hence, early diagnosis and effective treatment of gynecological cancer is the optimal means to reduce mortality^6–8^. Magnetic hyperthermia, as one of the new hyperthermia methods, shows good prospects for gynecological diseases and cancer treatment due to the characteristics of targeting, simple administration, reduced dosage, and minimal side effects^9–13^. During magnetic hyperthermia treatment, the magnetic nanoparticles are usually injected or targeted into the tumor lesion area, and the nanoparticles are heated by applying an alternating magnetic field (AMF). As a result, the tumor cells are destroyed while normal cells remain alive^14–17^. Most recently, the application of biomedical magnetic nanoparticles in gynecological tumor hyperthermia has received extensive attention, attributed to a series of features including good biosafety, surface modification, special *in-vitro* properties, and unique magnetism^18–20^.

Magnetic iron oxide nanoparticles, one of the significant biomaterials among novel biofunctional nanoparticles are widely considered the most suitable magnetic hyperthermia agents owing to their high saturation magnetization (*M*_s_)^21–24^. However, high synthesis process cost and weak thermal conversion efficiency restrict their extensive usage. Recently, the application of tunable nanomaterials is also becoming more widespread, and there are many mature preparation methods including sol-gel synthesis, hydrothermal reaction, sonochemical reaction and laser pyrolysis^25–29^. Generally, the traditional method is thermal decomposition of iron salts, which needs more energy and higher cost^30^. Hydrothermal synthesis, one of the preferred solutions to prepare biomedical Fe_3_O_4_ nanomaterials owing to the low cost and feasibility, while it can effectively regulate the size of nanomaterials by controlling hydrothermal time or reactant concentration^31–33^. In addition, nanorod materials have excellent magnetic properties due to their adjustable aspect ratio and strong anisotropy, thereby improving the heating efficiency of materials^8,34^.

Based on the foundation of enhanced magnetic hyperthermia by adjusting the aspect ratio of Fe_3_O_4_ nanorods (NRs), while hydrophilic graphene oxide (GO) was chosen as the counterpart for avoiding aggregation between Fe_3_O_4_ NRs. Hence, the different sizes of Fe_3_O_4_ NRs were synthesized by a simple hydrothermal method with different reaction times and post-annealing method at 340 °C. The *M*_s_ value of hydrophilic Fe_3_O_4_ NRs-GO with a length of 350 nm reaches the largest (60 emu/g). While it shows excellent magnetic hyperthermia performance with large SAR of 1045 W/g for a concentration of 0.2 mg/mL at 360 kHz, 308 Oe. Moreover, as a magnetic hyperthermia agent, Fe_3_O_4_ NRs can effectively slow the growth rate of gynecological cancer in nude mice. These results provide a feasible research basis for the application of high-performance magnetic hyperthermia agent in clinical practice.

## Experiment

### Synthesis of Fe_3_O_4_ NRs

Different sizes of Fe_3_O_4_ NRs were fabricated by a one-step hydrothermal method and reduction reaction^35^. First, 6.0 mM FeCl_3_ · 6H_2_O was added to 60 mL of deionized water and stirring continued for 0.5 h to form a homogeneous solution, then transferred to a Teflon-lined stainless-steel autoclave and reacted at 100 °C for 4, 6, and 10 h. The β-FeOOH precursors were collected by centrifugation and washed with deionized water and alcohol three times, then dried at 60 °C. Second, 20 mg of different sized β-FeOOH NRs was uniformly dispersed in 6 mL trioctylamine, then 200 μL oleic acid was added to the mixture and kept stirring for 2 h, respectively. Then the yellow gelatinous mixture was collected by centrifugation at 7500 rpm for 10 min. Finally, under the flow of mixed gas of 95% Ar and 5% H_2_ gas, the as-prepared precursors were transferred to a tube furnace for the reduction reaction at 340 °C and kept for 2 h, then naturally cooled to room temperature. The different sizes of Fe_3_O_4_ NRs were collected though centrifuging, washed with hexane, and dried at 60 °C. The 460, 350, and 250 nm Fe_3_O_4_ NRs correspond to different hydrothermal times of 4, 6, and 10 h, respectively.

### Synthesis of Fe_3_O_4_ NRs-GO

First, the as-prepared Fe_3_O_4_ NRs was dissolved in chloroform at a concentration of 10 mg/mL, and 5 mg/mL aqueous solution of graphene oxide (GO) was prepared. Then octadecylamine (20 mg) and chloroform (1 mL) were fully dissolved by ultrasound for 1-2 min, then Fe_3_O_4_ NRs solution (100 μL), deionized water (4 mL) and GO (1 mL) were added to further ultrasound for 30 min. Finally, the obtained solution was placed on a shaker for 6 h at 55 °C to remove chloroform, then the Fe_3_O_4_ NRs-GO dispersion system was prepared.

### Materials characterization

The phase structure, morphology, and chemical composition on the surface of the Fe_3_O_4_ NRs were analyzed by X-ray diffraction (XRD) patterns (X’pert Pro Philips), scanning electron microscope (SEM, Hitachi S-4800), transmission electron microscope (TEM, Tecnai G2 F30), and X-ray photoelectron spectroscopy (XPS, Kratos AXIS Ultra), respectively. Vibrating sample magnetometer (VSM Model EV9, MicroSense, LLC) and SQUID systems (MPMSXL-7) were carried out to measure hysteresis curves and zero field cooled (ZFC) and field cooled (FC) magnetization plots. Further, the hydrodynamic diameters and zeta potential of samples were measured on a Malvern Zetasizer Nano-ZS. The magnetic heating properties of Fe_3_O_4_ NRs were analyzed by a radiofrequency heating machine (EASYHEAT-5060, Ambrell), the specific absorption rate (SAR) value was calculated based on the following equation: ${\rm{SAR}}={\rm{C}}\frac{\Delta T}{\Delta t}\left(\frac{1}{{m}_{Fe}}\right)$, where $\frac{\Delta T}{\Delta t}$ is the initial slope of the temperature versus time curve, C is the specific heat capacity of water, and *m*_*Fe*_ is the weight fraction of Fe in solution.

### *In vivo* experimental sequences

Sixteen female 6–8 weeks old nude mice, weighing about 20 g, purchased from Beijing Weitong lihua Experimental Animal Technology Co., Ltd, were kept in a sterile environment. Four mice were placed in each autoclaved cage and the animals were fed with a special diet, given water, and kept at an appropriate temperature. 0.1 mL of cancer cell suspension (about 1*107 cells) was injected subcutaneously into the right lower extremities of mice to induce tumors and observe the growth of mice. Then mice were randomly divided into 4 groups with a tumor nodule volume as long as about 80 mm^3^, and 4 mice in each group. Nude mice in the experimental group were injected subcutaneously with 0.1 mL (Fe concentration of about 1.2 mg/mL) of Fe_3_O_4_ NRs-GO-AFM and Fe_3_O_4_ NRs-GO, respectively. While in the control group, nude mice were injected with 0.1 mL of PBS buffer. Before treatment, the initial tumor volume of each nude mouse was measured and recorded (Volume = length * width * Width/2)^36,37^. Then three groups of nude mice were placed under a magnetic field of 308 Oe for 10 min. Finally, nude mice were sacrificed after 20 days, and tumor volume changes of each nude mouse were measured every 2 days before euthanasia.

## Results and discussion

**Figure 1:**
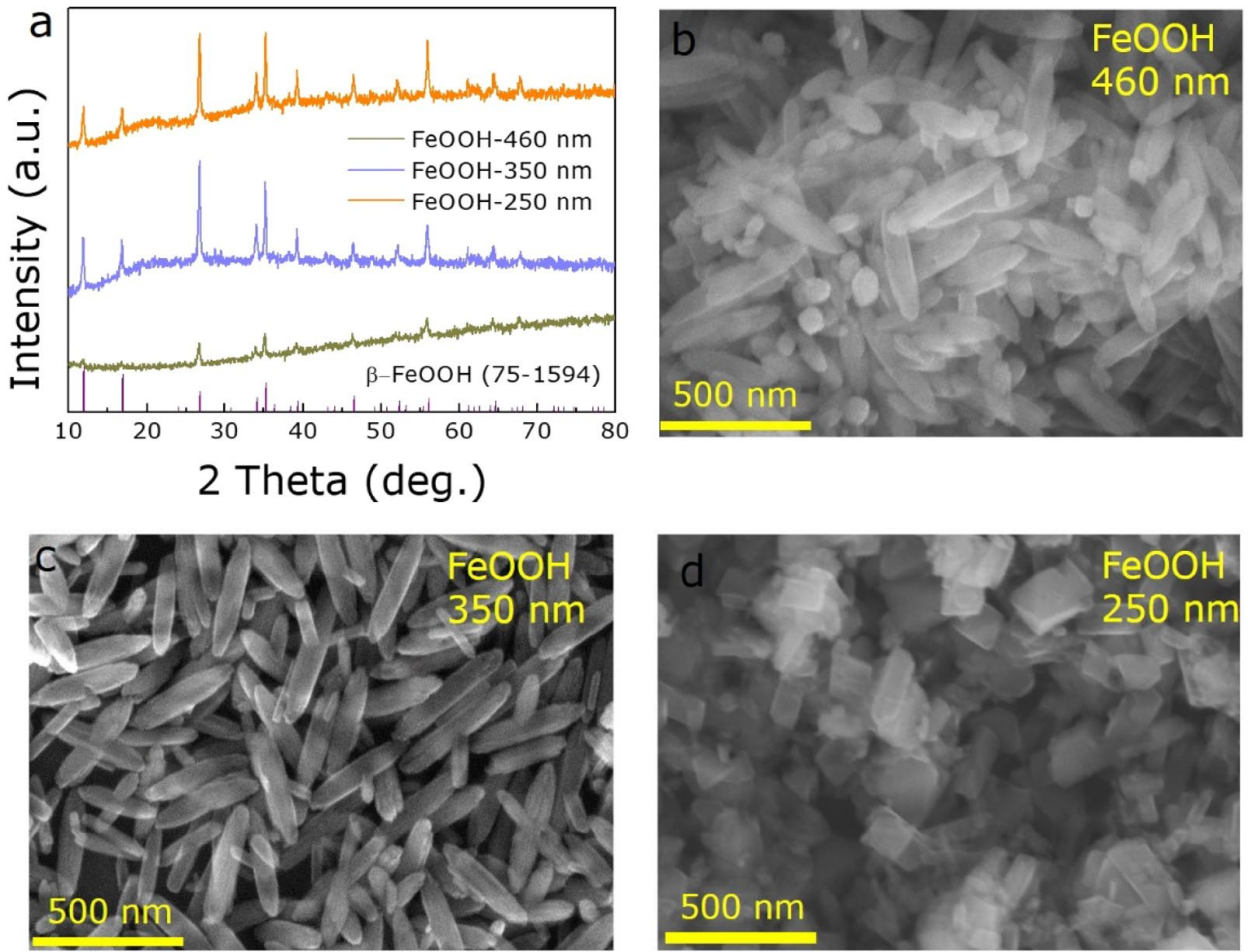
The structure and morphology of FeOOH precursors. (**a**) XRD patterns of FeOOH. (**b**–**d**)_._ SEM images of 460, 350 and 250 nm FeOOH NRs, respectively.

Different size of FeOOH NRs were prepared by a simple hydrothermal method with various reaction times. Figure 1a shows the X-ray diffraction (XRD) patterns of the prepared precursors of FeOOH NRs, all the diffraction peaks are well indexed to the β-FeOOH (JCPPS no. 75-1594). Figure 1b–d illustrate the scanning electron microscope (SEM) images of FeOOH NRs with lengths of 460 nm, 350 nm, and 250 nm, respectively, which correspond to the experimental conditions of 4 h, 6 h, and 10 h hydrothermal times, respectively.

**Figure 2:**
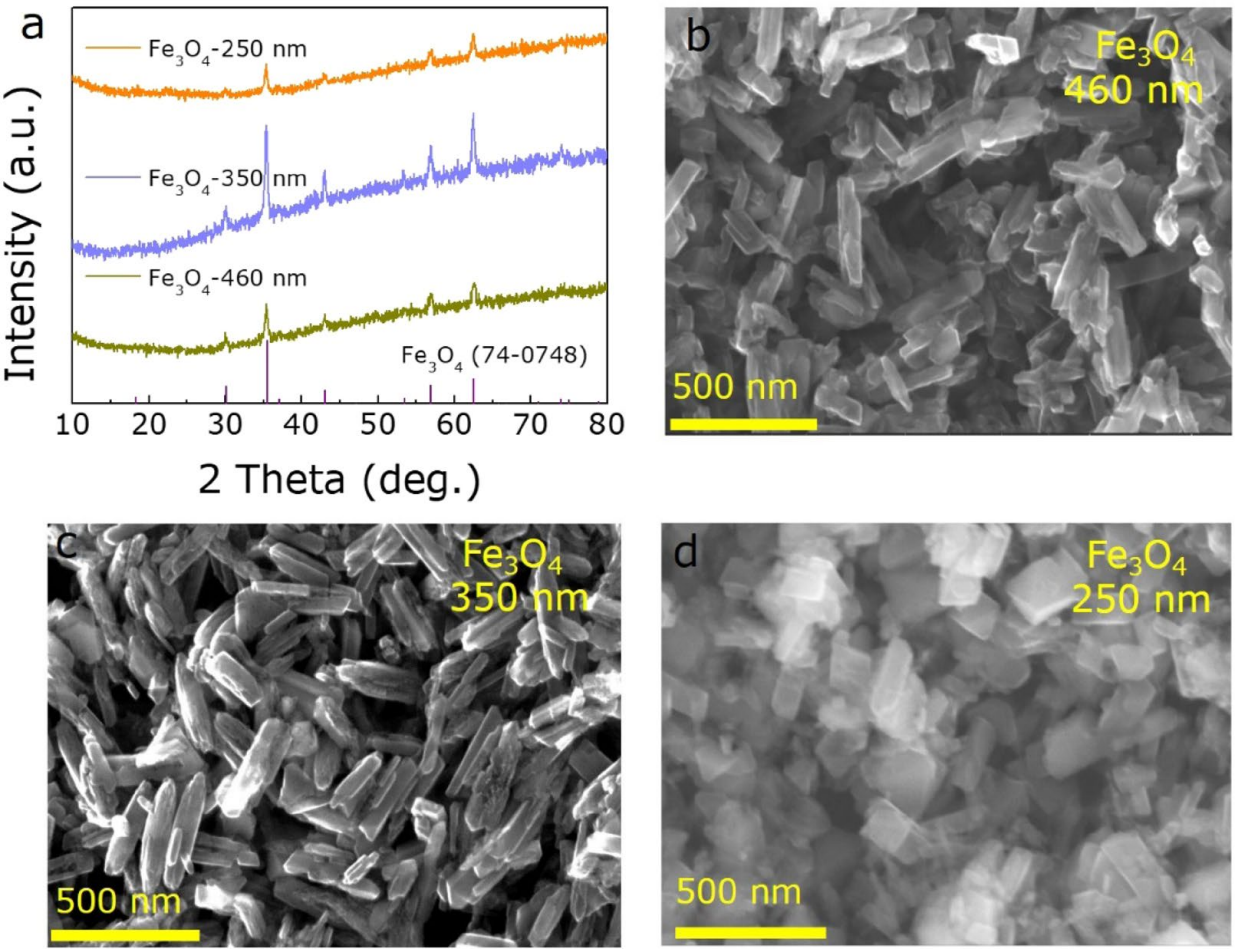
The structure and morphology of Fe_3_O_4_ samples. (**a**) XRD patterns of Fe_3_O_4_. (**b**–**d**) SEM images of 460 nm, 350 nm and 250 nm Fe_3_O_4_ NRs, respectively.

**Figure 3:**
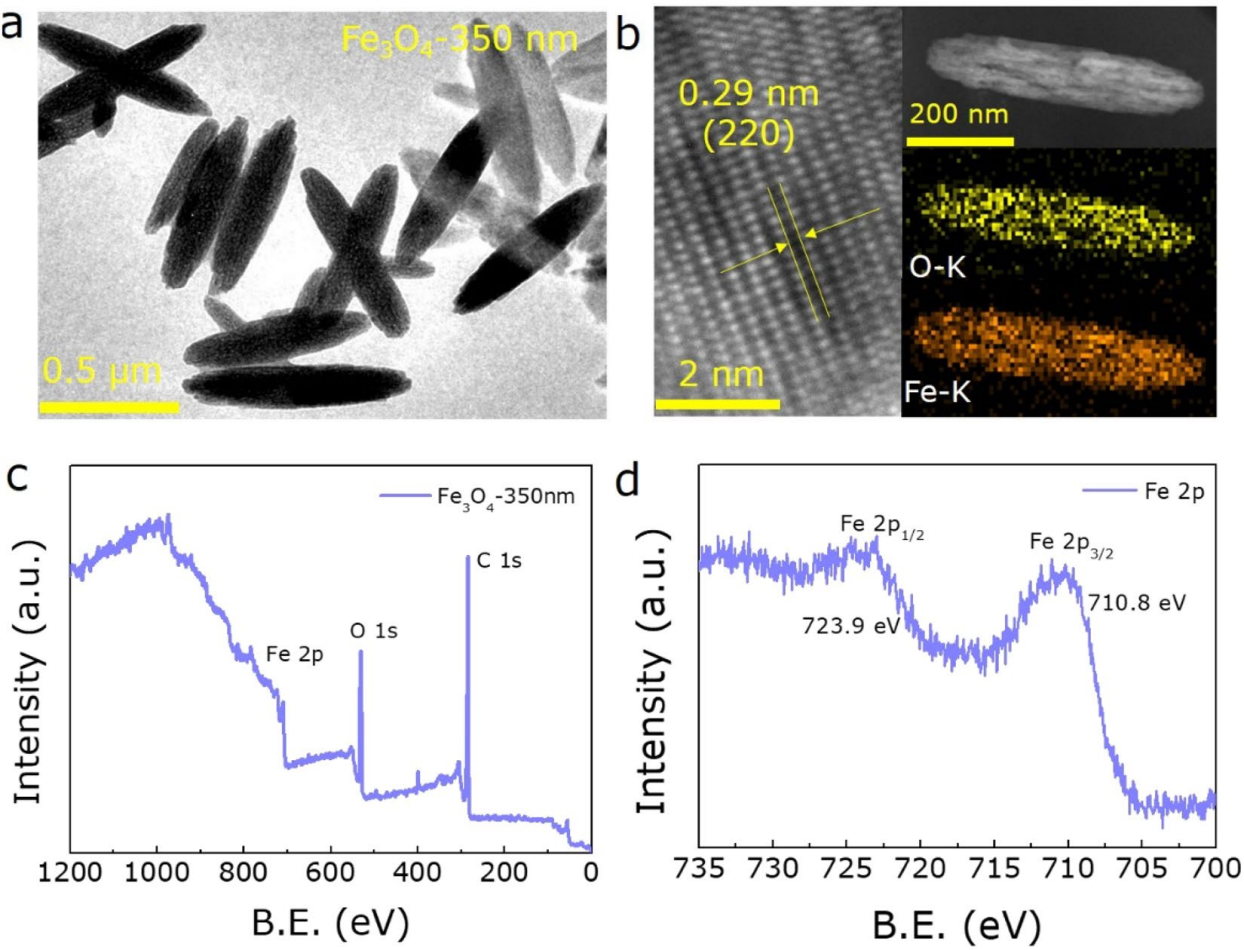
The detailed characterizations of 350 nm Fe_3_O_4_ NRs sample. (**a**) TEM image. (**b**) HRTEM image and element mappings of 350 nm Fe_3_O_4_ NRs. XPS spectra of (**c**) wide and (**d**) Fe 2p spectrum for 350 nm Fe_3_O_4_ NRs.

After annealing at 340 °C for 2 h in a mixed gas of 95% Ar and 5% H_2_, FeOOH precursors were reduced to Fe_3_O_4_ NRs as confirmed by XRD patterns (Fig. 2a). All the diffraction patterns of three samples correspond to the Fe_3_O_4_ (JCPPS no. 74-0748), where no impurity phase or second phase (e.g. FeO, Fe, or FeOOH) are produced, indicating that the FeOOH are completely reduced to Fe_3_O_4_ after calcination. Besides, the average size and morphology of Fe_3_O_4_ NRs are basically unchanged compared to the FeOOH NRs precursors, as described by SEM and TEM images shown in Figs. 2b–d and 3a, where a slight surface roughness change is possibly caused by the incomplete reaction of oleic acid as the capping agent^12,31^.

For the 350 nm Fe_3_O_4_ NRs, the high-resolution TEM (HRTEM) image and EDX mapping analysis were also acquired as shown in Fig. 3b. The clear lattice fringes with *d*-spacing of 0.29 nm, correspond to (220) crystal plane of Fe_3_O_4_, demonstrating that the 350 nm Fe_3_O_4_ NRs has excellent crystallinity. Additionally, the EDX mapping analysis illustrates that the Fe and O atoms are uniformly distributed in the Fe_3_O_4_ NRs matrix. Furthermore, X-ray photoelectron spectroscopy (XPS) was performed to determine the surface chemical composition of the 350 nm Fe_3_O_4_ NRs sample. As seen in the survey scan (Fig. 3c), Fe, O, and C elements coexist, corresponding to the chemical composition of Fe_2_O_3_. Figure 3d further shows the Fe 2p spectrum, where the binging energy peaks at 723.9 and 710.8 eV match well with the Fe 2p_1/2_ and Fe 2p_3/2_ levels, respectively, confirming the existence of pure Fe_3_O_4_. The typical satellite peaks at 729.5 and 719.0 eV have not been identified, which further illustrates the full reduction of β-FeOOH precursor and high purity of Fe_3_O_4_ NRs^38,39^.

**Figure 4:**
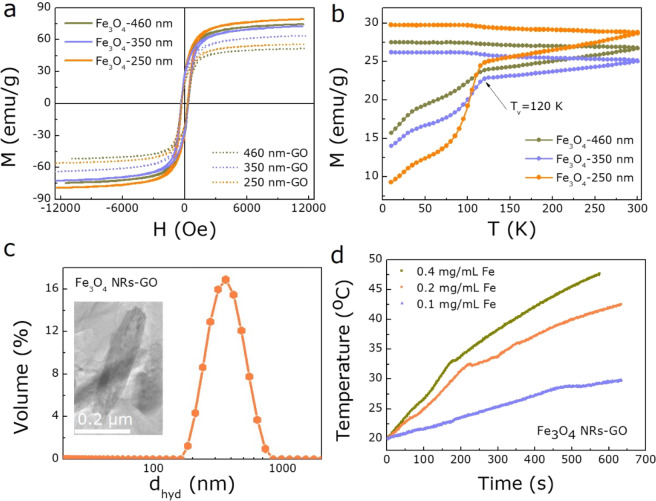
The magnetothermal performances of Fe_3_O_4_ NRs. The magnetization hysteresis loops (**a**) and temperature-dependent ZFC-FC curves (**b**) of all Fe_3_O_4_ NRs. (**c**) Hydrodynamic size of 350 nm Fe_3_O_4_ NRs-GO. The inset: The morphology of Fe_3_O_4_ NRs-GO. (**d**) Heating curves for 350 nm Fe_3_O_4_ NRs at the concentration of 0.1, 0.2, and 0.4 mg/mL.

Subsequently, a vibrating sample magnetometer (VSM) was used to estimate the magnetic properties of Fe_3_O_4_ NRs. Figure 4a shows the room temperature *M(H)* hysteresis loop of samples, the saturation magnetization (*M*_s_) of 460, 350, and 250 nm Fe_3_O_4_ NRs are measured to be 72, 71 and 77 emu/g, respectively. Almost equal coercivity *(H*_c_) and remanence (*M*_r_) were obtained for three samples. However, the *M*_s_ value is not changed significantly with the change of size for Fe_3_O_4_ NRs, and lower than that of bulk Fe_3_O_4_ (90 emu/g) at room temperature, which is possibly caused by the tiny uncompensated surface spin or the disordered surface microstructures^40^. The ZFC and FC magnetization plot of three Fe_3_O_4_ NRs samples were measured to further confirm the phase structure and crystallinity of Fe_3_O_4_. The Verwey transition temperature (T_v_) is detected at 120 K as shown in Fig. 4b, which is one of the criteria for judging Fe_3_O_4_^41,42^_,_ in accordance with the above XRD, XPS results.

**Figure 5:**
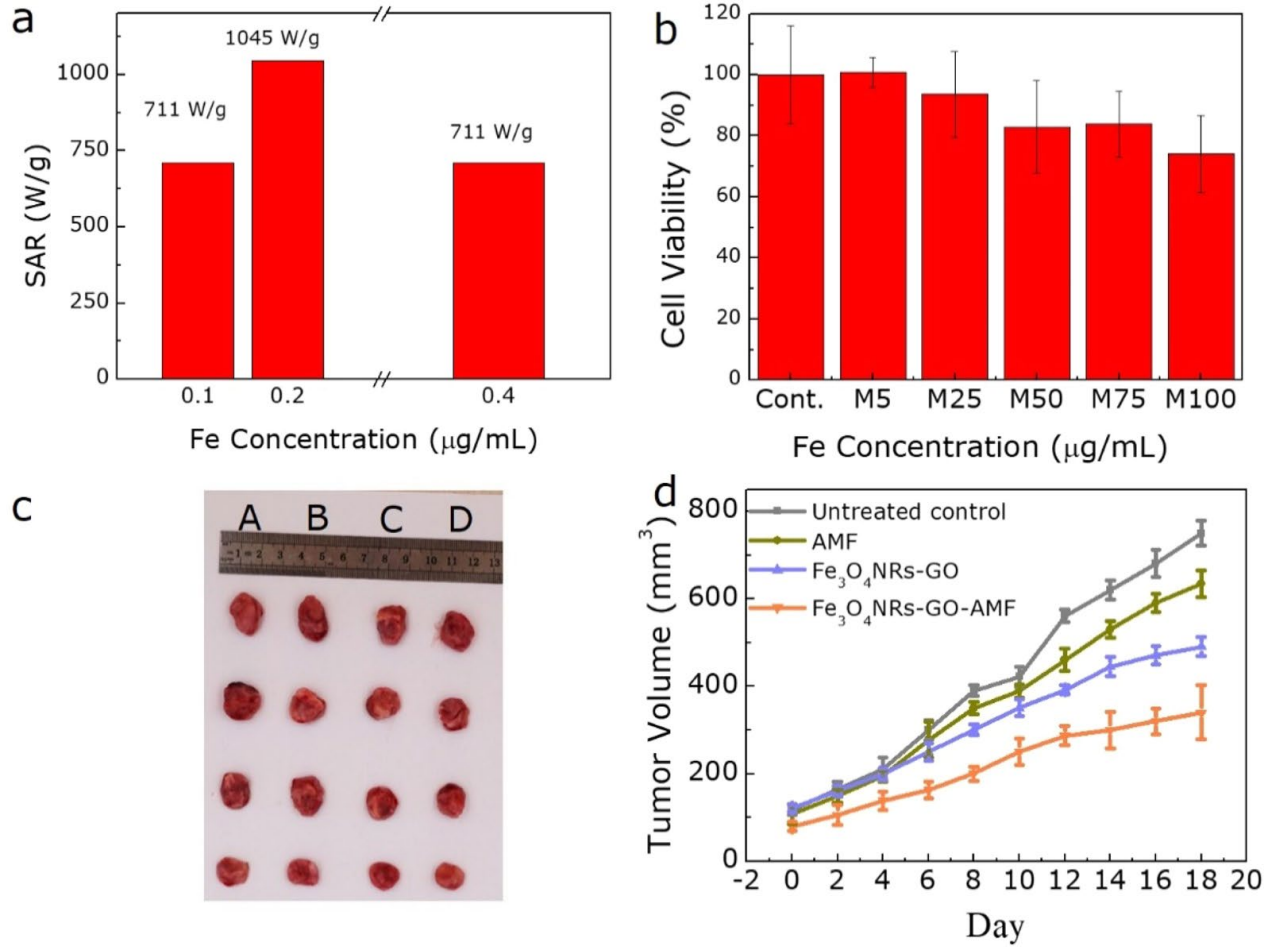
(**a**) The SAR values for 350 nm Fe_3_O_4_ NRs at the concentration of 0.1, 0.2, and 0.4 mg/mL. (**b**) Biocompatibility of 350 nm Fe_3_O_4_ NRs. (**c**) Photograph of nude mice xenografted with gynecological cancer cells before treatment and 18 days after treatment with the untreated control, 350 nm Fe_3_O_4_ NRs hyperthermia, respectively and (**d**) the corresponding plot of tumor volume versus days after treatment.

To further understand the hyperthermia performance of samples, the transformation of the aqueous phase was first performed to evenly disperse the Fe_3_O_4_ NRs in water^43^. Then the magnetic properties of as-prepared samples were again analyzed as shown in Fig. 4a, the *M*_s_ of 350 nm Fe_3_O_4_ NRs-GO reaches a maximum of 60 emu/g, suggesting it has the best hyperthermia performance^44^. Besides, Fig. 4c shows that the Fe_3_O_4_ NRs-GO dispersion is without obvious aggregation and stability, which is demonstrated by hydrodynamic diameter of 370 nm and zeta-potential of 50.1 mV, and the morphology of Fe_3_O_4_ NRs-GO as shown in the inset of Fig. 4c. Therefore, to study the effect of different concentrations of 350 nm Fe_3_O_4_ NRs on heating efficiency, the SAR value was measured and analyzed at 308 Oe and 360 KHz. Figure 4d shows the heating performance as a function of temperature for 350 nm Fe_3_O_4_ NRs with different concentrations of 0.1, 0.2, and 0.4 mg/mL Fe, where a shorter time of 10 min is observed to easily reach 42 °C for 0.2 mg/mL Fe_3_O_4_ NRs, indicating that it is suitable for high temperature magnetothermal application. Further, the heating rate significantly accelerates as the concentration of Fe increases to 0.4 mg/mL. Correspondingly, the sample with 0.2 mg/mL Fe achieves the largest SAR value of 1045 W/g, superior to other concentration samples as shown in Fig. 5a, demonstrating the excellent magnetic hyperthermia performance of Fe_3_O_4_ NRs, further providing a powerful basis for the development and utilization of a magnetic hyperthermia agent. The reduction in the heating efficiency with increasing concentration of 0.4 mg/mL Fe maybe due to the higher aggregation of the Fe_3_O_4_ NRs, which tends to negatively affect their heating capacity^35,45^. In addition, Fe_3_O_4_ NRs have a low cytotoxic effect for gynecological cancer cells with the concentration of Fe from 5 to 100 μg/mL (Fig. 5b), revealing the good biocompatibility of biomedical Fe_3_O_4_ NRs. Therefore, further study on cell magnetic hyperthermia was performed. All protocols for animal research conformed to the Guide for the Care and Use of Laboratory Animals, and approved by the Lanzhou University Ethics Committee. All the *in vivo* studies were conducted according to guidelines that were approved by the Institutional Animal Care and Use Committee of the Tsinghua University. After 10 min of magnetic hyperthermia with Fe_3_O_4_ NRs-GO, the growth rate of tumors is significantly slowed by tracking the growth of tumors in nude mice for 18 days (Fig. 5c,d). These results indicate that Fe_3_O_4_ NRs can be used as a high-performance magnetic hyperthermia agent to effectively limit the growth of gynecological cancer.

## Conclusions

Highly crystalline and tunable size Fe_3_O_4_ NRs have been successfully synthesized by reducing β-FeOOH precursor at 340 °C, while their magnetic and inductive heating properties have been researched. At room temperature, 460, 350, and 250 nm Fe_3_O_4_ NRs could display relatively high *M*_s_ values, where the 350 nm Fe_3_O_4_ NRs exhibits a large SAR value of 1045 W/g at low magnetic field (308 Oe), attributing to the high *M*_s_ and effective anisotropy of nanorod materials. As a result, it reveals good biocompatibility, which is a precondition for magnetic hyperthermia investigation on cancers in the future. Furthermore, as a magnetic hyperthermia agent, the as-prepared Fe_3_O_4_ NRs could effectively alleviate the growth of gynecological cancer in the nude mice model, providing an effective approach for the treatment of clinical gynecological cancer in the future.

## Data Availability

The datasets generated during and/or analyzed during the current research are available from the corresponding author on reasonable request.

## References

[1] Sailor, M. J. & Park, J. H. Hybrid nanoparticles for detection and treatment of cancer. *Adv. Mater.* **24**, 3779–3802 (2012). 22610698 10.1002/adma.201200653 PMC3517011

[2] Liu, X. L. *et al*. Innovative magnetic nanoparticle platform for magnetic resonance imaging and magnetic fluid hyperthermia applications. *Curr. Opin. Chem. Eng.* **4**, 38–46 (2014).

[3] Jun, Y.-w *et al*. Nanoscaling laws of magnetic nanoparticles and their applicabilities in biomedical sciences. *Acc. Chem. Res.* **41**, 179–189 (2008). 18281944 10.1021/ar700121f

[4] Wu, W. *et al*. Recent progress on magnetic iron oxide nanoparticles: synthesis, surface functional strategies and biomedical applications. *Sci. Technol. Adv. Mat.* **16**, 023501 (2015). 10.1088/1468-6996/16/2/023501 PMC5036481 27877761

[5] Gandia, D. *et al*. Unlocking the Potential of Magnetotactic Bacteria as Magnetic Hyperthermia Agents. *Small* **15**, 1902626 (2019). 10.1002/smll.201902626 31454160

[6] Barreto, J. A. *et al*. Nanomaterials: applications in cancer imaging and therapy. *Adv. Mater.* **23**, H18–H40 (2011). 21433100 10.1002/adma.201100140

[7] Pankhurst, Q. *et al*. Progress in applications of magnetic nanoparticles in biomedicine. *J. Phys. D. Appl. Phys* **42**, 224001 (2009).

[8] Mohapatra, J. *et al*. Iron oxide nanorods as high-performance magnetic resonance imaging contrast agents. *Nanoscale* **7**, 9174–9184 (2015). 25849780 10.1039/c5nr00055f

[9] Pankhurst, Q. A. *et al*. Applications of magnetic nanoparticles in biomedicine. *J. Phys. D Appl. Phys.* **36**, R167 (2003).

[10] Barreto, J. A. *et al*. Colloidal stability and 64 Cu labeling of iron oxide nanoparticles bearing different macrocyclic ligands. *New J. Chem.* **35**, 2705–2712 (2011).

[11] Lim, E.-K. *et al*. Nanomaterials for theranostics: recent advances and future challenges. *Chem. Rev.* **115**, 327–394 (2014). 25423180 10.1021/cr300213b

[12] Geng, S. *et al*. Anisotropic magnetite nanorods for enhanced magnetic hyperthermia. *Chem. Asian J.* **11**, 2996–3000 (2016). 27615802 10.1002/asia.201601042

[13] Das, R. *et al*. Magnetically tunable iron oxide nanotubes for multifunctional biomedical applications. *J. Alloy. Comp* **789**, 323–329 (2019).

[14] Rosensweig, R. E. Heating magnetic fluid with alternating magnetic field. *J. Magn. Magn. Mater.* **252**, 370–374 (2002).

[15] Glöckl, G. *et al*. The effect of field parameters, nanoparticle properties and immobilization on the specific heating power in magnetic particle hyperthermia. *J. Phys. Condens. Mat* **18**, S2935 (2006).

[16] Martinez-Boubeta, C. *et al*. Learning from nature to improve the heat generation of iron-oxide nanoparticles for magnetic hyperthermia applications. *Sci. Rep* **3**, 1652 (2013). 23576006 10.1038/srep01652 PMC3622918

[17] Huong, P. T. L. *et al*. Magnetic iron oxide-carbon nanocomposites: Impacts of carbon coating on the As (V) adsorption and inductive heating responses. *J. Alloy. Comp* **739**, 139–148 (2018).

[18] Petros, R. A. *et al*. Strategies in the design of nanoparticles for therapeutic applications. *Nat. Rev. Drug Discov.* **9**, 615 (2010). 20616808 10.1038/nrd2591

[19] Mehdaoui, B. *et al*. Optimal size of nanoparticles for magnetic hyperthermia: a combined theoretical and experimental study. *Adv. Funct. Mater.* **21**, 4573–4581 (2011).

[20] Nemati, Z. *et al*. Iron oxide nanospheres and nanocubes for magnetic hyperthermia therapy: a comparative study. *J. Elec. Mater* **46**, 3764–3769 (2017).

[21] Yu, J. *et al*. Multifunctional Fe_5_C_2_ nanoparticles: a targeted theranostic platform for magnetic resonance imaging and photoacoustic tomography‐guided photothermal therapy. *Adv. Mater.* **26**, 4114–4120 (2014). 24677251 10.1002/adma.201305811

[22] Gao, J. *et al*. Multifunctional magnetic nanoparticles: design, synthesis, and biomedical applications. *Accounts Chem. Res.* **42**, 1097–1107 (2009). 10.1021/ar9000026 19476332

[23] Lee, N. *et al*. Iron oxide based nanoparticles for multimodal imaging and magnetoresponsive therapy. *Chem. Rev.* **115**, 10637–10689 (2015). 26250431 10.1021/acs.chemrev.5b00112

[24] Nemati, Z. *et al*. Improving the heating efficiency of iron oxide nanoparticles by tuning their shape and size. *J. Phys. Chem. C* **122**, 2367–2381 (2018).

[25] Bao, L. *et al*. Colloidal synthesis of magnetic nanorods with tunable aspect ratios. *J. Mater. Chem.* **22**, 7117–7120 (2012).

[26] Sun, H. *et al*. Solvothermal synthesis of tunable electroactive magnetite nanorods by controlling the side reaction. *J. Phys. Chem. C* **116**, 5476–5481 (2012).

[27] Deng, Y. *et al*. Preparation of magnetic polymeric particles via inverse microemulsion polymerization process. *J. Magn. Magn. Mater.* **257**, 69–78 (2003).

[28] Mukh-Qasem, R. A. *et al*. Sonochemical synthesis of stable hydrosol of Fe_3_O_4_ nanoparticles. *J.Colloid Interf. Sci* **284**, 489–494 (2005). 10.1016/j.jcis.2004.10.073 15780286

[29] Nemati, Z. *et al*. Enhanced magnetic hyperthermia in iron oxide nano-octopods: size and anisotropy effects. *J. Phys. Chem. C* **120**, 8370–8379 (2016).

[30] Lee, J.-H. *et al*. Exchange-coupled magnetic nanoparticles for efficient heat induction. *Nat. nanotechnol* **6**, 418 (2011). 21706024 10.1038/nnano.2011.95

[31] Si, J.-C. *et al*. Solvothermal synthesis of tunable iron oxide nanorods and their transfer from organic phase to water phase. *CrystEngComm* **16**, 512–516 (2014).

[32] Khurshid, H. *et al*. Mechanism and controlled growth of shape and size variant core/shell FeO/Fe_3_O_4_ nanoparticles. *Nanoscale* **5**, 7942–7952 (2013). 23857290 10.1039/c3nr02596a

[33] Nemati, Z. *et al*. Core/shell iron/iron oxide nanoparticles: are they promising for magnetic hyperthermia? *RSC Adv* **6**, 38697–38702 (2016).

[34] Kopwitthaya, A. *et al*. Biocompatible PEGylated gold nanorods as colored contrast agents for targeted *in vivo* cancer applications. *Nanotechnology* **21**, 315101 (2010). 20622303 10.1088/0957-4484/21/31/315101

[35] Das, R. *et al*. Tunable high aspect ratio iron oxide nanorods for enhanced hyperthermia. *J. Phys. Chem. C* **120**, 10086–10093 (2016).

[36] Euhus, D. M. *et al*. Tumor measurement in the nude mouse. *J. Surgi. Oncol* **31**, 229–234 (1986). 10.1002/jso.2930310402 3724177

[37] Tomayko, M. M. *et al*. Determination of subcutaneous tumor size in athymic (nude) mice. *Cancer Chemoth. Pharm* **24**, 148–154 (1989). 10.1007/BF00300234 2544306

[38] Liu, S. *et al*. Polarity and surface structural evolution of iron oxide films. *RSC Adv* **2**, 9938–9943 (2012).

[39] Radu, T. *et al*. X-ray photoelectron spectroscopic characterization of iron oxide nanoparticles. *Appl. Surf. Sci.* **405**, 337–343 (2017).

[40] Sun, S. *et al*. Size-controlled synthesis of magnetite nanoparticles. *J. Am. Chem. Soc.* **124**, 8204–8205 (2002). 12105897 10.1021/ja026501x

[41] Garaio, E. *et al*. A multifrequency eletromagnetic applicator with an integrated AC magnetometer for magnetic hyperthermia experiments. *Meas. Sci. Technol.* **25**, 115702 (2014).

[42] Poddar, P. *et al*. First-order metal-insulator transition and spin-polarized tunneling in Fe_3_O_4_ nanocrystals. *Phys. Rev. B* **65**, 172405 (2002).

[43] Liu, X. *et al*. Ultrasonication-Triggered Ubiquitous Assembly of Magnetic Janus Amphiphilic Nanoparticles in Cancer Theranostic Applications. *Nano Lett*. **2019**. 10.1021/acs.nanolett.9b01524 31140281

[44] Conde-Leboran, I. *et al*. A single picture explains diversity of hyperthermia response of magnetic nanoparticles. *J. Phys. Chem. C* **119**, 15698–15706 (2015).

[45] Andreu, I. *et al*. Nano-objects for addressing the control of nanoparticle arrangement and performance in magnetic hyperthermia. *ACS Nano* **9**, 1408–1419 (2015). 25658023 10.1021/nn505781f

